# Lateral mobility of L-type calcium channels in synaptic terminals of retinal bipolar cells

**Published:** 2013-01-07

**Authors:** Wallace B. Thoreson, Aaron J. Mercer, Karlene M. Cork, Robert J. Szalewski

**Affiliations:** 1 Departments of Ophthalmology & Visual Sciences, University of Nebraska Medical Center, Omaha, NE; 2 Pharmacology & Experimental Neuroscience, University of Nebraska Medical Center, Omaha, NE; 3 Department of Molecular & Integrative Physiology, University of Michigan, Ann Arbor, MI

## Abstract

**Purpose:**

Efficient and precise release of glutamate from retinal bipolar cells is ensured by the positioning of L-type Ca^2+^ channels close to release sites at the base of the synaptic ribbon. We investigated whether Ca^2+^ channels at bipolar cell ribbon synapses are fixed in position or capable of moving in the membrane.

**Methods:**

We tracked the movements of individual L-type Ca^2+^ channels in bipolar cell terminals after labeling channels with quantum dots (QDs) attached to α_2_δ_4_ accessory Ca^2+^ channel subunits via intermediary antibodies.

**Results:**

We found that individual Ca^2+^ channels moved within a confined domain of 0.13–0.15 μm^2^ in bipolar cell terminals, similar to ultrastructural estimates of the surface area of the active zone beneath the ribbon. Disruption of actin expanded the confinement domain indicating that cytoskeletal interactions help to confine channels at the synapse, but the relatively large diffusion coefficients of 0.3–0.45 μm^2^/s suggest that channels are not directly anchored to actin. Unlike photoreceptor synapses, removing membrane cholesterol did not change domain size, indicating that lipid rafts are not required to confine Ca^2+^ channels at bipolar cell ribbon synapses.

**Conclusions:**

The ability of Ca^2+^ channels to move within the presynaptic active zone suggests that regulating channel mobility may affect release from bipolar cell terminals.

## Introduction

The transmission of light responses to retinal ganglion and amacrine cells is regulated by the entry of Ca^2+^ through L-type Ca^2+^ channels in the synaptic terminals of retinal bipolar cells [1,2]. Release from bipolar cells can be triggered by the opening of only a handful of Ca^2+^ channels [3,4], and opening a single Ca^2+^ channel is sufficient to trigger vesicle fusion in rod bipolar cells [5]. This highly efficient coupling between channel opening and release is ensured by the clustering of Ca^2+^ channels near the synaptic ribbon [6,7], a protein structure that tethers vesicles near release sites [8,9]. Tight coupling between Ca^2+^ channel opening and release has been observed at other ribbon and non-ribbon synapses [10-14]. The clustering of Ca^2+^ channels at the active zone in ribbon and non-ribbon synapses involves direct and indirect interactions between Ca^2+^ channels and multiple proteins such as bassoon, RIBEYE, RIM, CAST, and CaBP4 [12,15-18]. Despite these many interacting protein partners, Ca^2+^ channels in photoreceptor terminals are not fixed in position and can move with relative freedom within a small patch of membrane beneath the ribbon [19]. In the present study, we asked whether L-type Ca^2+^ channels clustered at bipolar cell ribbons are also capable of moving in the presynaptic membrane. To study Ca^2+^ channel movements, we labeled individual L-type Ca^2+^ channels by attaching a photostable quantum dot (QD) to the α_2_δ_4_ accessory Ca^2+^ channel subunit through intermediary primary and secondary antibodies [19]. We targeted the α_2_δ subunit because its large extracellular domain makes it accessible to antibodies applied in living tissue. α_2_δ_4_ is the principal α_2_δ subunit in photoreceptors and is strongly expressed in the inner plexiform layer (IPL) suggesting it is also an accessory subunit at L-type Ca^2+^ channels in bipolar cell terminals [19,20].

The results of this study showed that Ca^2+^ channels move within a small confined domain in bipolar cell terminals similar to the size of the active zone beneath the ribbon determined from ultrastructural analysis [21]. Disrupting the actin cytoskeleton increased the size of this confinement domain, but cholesterol depletion did not, suggesting that interactions with the cytoskeleton, but not lipid rafts, help to confine channels at bipolar cell synapses. The finding that presynaptic Ca^2+^ channels are mobile suggests that, as in photoreceptors [22], changes in channel mobility might affect synaptic release at the bipolar cell ribbon synapse.

## Methods

### Animal care and use

Experimental procedures were approved by the University of Nebraska Medical Center Institutional Animal Care and Use Committee. Aquatic tiger salamanders (*Ambystoma tigrinum*, 18–25 cm long; Kons Scientific, Germantown, WI; Charles Sullivan Co., Nashville, TN) were maintained on a 12 h:12 h light-dark cycle and were sacrificed 1–2 h after the beginning of subjective night. Salamanders were decapitated with heavy shears and immediately pithed.

### Western blot analysis

*Ambystoma tigrinum* eyes were dissected, and two whole retinas were collected in ice-cold radioimmunoprecipitation buffer (containing, in mM, 25 Tris-HCl pH 7.6, 150 mM NaCl, 1% NP-40, 1% sodium deoxycholate and 0.1% sodium dodecyl sulfate [SDS]) and resolved on 10% Bis-Tris polyacrylamide gels (Invitrogen, Carlsbad, CA). Gels were transferred to nitrocellulose membranes and blotted with antibodies to α_2_δ_4_ (1:1000) with an anti-β-actin antibody serving as a housekeeping protein (1:10,000, Sigma-Aldrich, St. Louis, MO). Blots were visualized using horseradish peroxidase-conjugated secondary antibodies and SuperSignal West Pico Chemiluminescent Substrate (Pierce/Thermo, Waltham, MA).

### Retinal tissue preparation

After enucleation, each eye was opened along the ora serrata, and the cornea, lens, and vitreous body were removed. For experiments on retinal slices, the eyecup was sectioned into thirds, and a piece of eyecup was pressed vitreous side down onto a 5 × 10 mm piece of filter paper (type AAWP, 0.8 μm pores; Millipore, Billerica, MA). The filter paper and eyecup were then immersed in cold amphibian saline solution consisting of (in mM) 111 NaCl, 2.5 KCl, 1.8 CaCl_2_, 0.5 MgCl_2_, 10 HEPES, and 5 glucose (pH 7.8). The retinal pigment epithelium, choroid, and sclera were peeled away, leaving the retina adhered to the filter paper. The retina was sliced into 125 μm sections with a razor blade tissue chopper (Stoelting, Wood Dale, IL) fitted with a #121–6 razor blade (Ted Pella Inc., Redding, CA). Slices were separated from one another and rotated 90° to view the retinal layers under a water immersion objective.

For experiments on dissociated cells, a salamander retina was isolated and incubated in 0.5 mM Ca^2+^ amphibian extracellular saline solution containing 0.2 mg/ml cysteine and 10 U/ml papain (Sigma-Aldrich) for about 35 min at 20 °C. Tissue was washed twice in a low Ca^2+^ saline solution containing 1% BSA and 1 mg/ml DNase (Worthington Biochemicals, Lakewood, NJ) followed by two additional washes in low Ca^2+^ saline. To isolate individual neurons, retinal tissue was gently triturated using a fire-polished glass Pasteur pipette and plated onto 18 mm coverslips (Deckgläser/Thermo, Braunschweig, Germany) coated with Cell Tak (BD Biosciences, San Jose, CA).

### Quantum dot binding and imaging

QD attachment to individual Ca^2+^ channels at bipolar cell synapses was conducted as described previously for photoreceptors [19]. Briefly, retinal neurons were incubated with a primary rabbit anti-α_2_δ_4_ subunit antibody [23] (1:1,000 dilution for isolated cells and 1:500 for retinal slices) in standard 1.8 mM Ca^2+^ saline solution for 3 h at 4 °C. After incubation with the primary antibody, cells were washed 3× with standard amphibian saline solution, and then incubated with secondary biotinylated goat-anti-rabbit immunoglobulin G (1:2000; Sigma-Aldrich) for 1 h at 4 °C. In some experiments, the actin disruptor cytochalasin D (20 μM) or cholesterol-depleting agent methyl-β-cyclodextrin (MβCD; 10 mM) was included with the secondary antibody incubation. After this step, retinal tissue was washed another 3X and then incubated with streptavidin-coated 525 or 655 nm emission QDs (20 nM) for 15 min. Tissue was washed eight more times before visualization. As a control for baseline noise, we also visualized and quantified the movements of QDs immobilized in vacuum grease. Indirect immunofluorescence with fluorescein isothiocyanate (FITC)–conjugated secondary antibodies showed strong labeling by the α_2_δ_4_ antibody throughout the IPL where bipolar cell terminals reside [19]. Labeling with FITC or QDs was almost completely abolished by omitting the primary antibody or coincubating the primary antibody with the peptide used to develop the antibody [19].

To visualize QDs attached to dissociated bipolar cells, we used an IX71 inverted microscope (Olympus, Center Valley, PA) with a 60X, 1.45 NA oil-immersion objective (Olympus) and cooled EMCCD camera (ImageM-1K, Hamamatsu, Japan). Epifluorescence was provided by an X-Cite 120Q light source (Lumen Dynamics Group Inc., Mississauga, Canada) with a FITC filter cube and Model D122 shutter (UniBlitz, Rochester, NY). Videos were captured using MetaMorph imaging software (Molecular Devices, Sunnyvale, CA). For retinal slices, QDs were visualized through a 60X, 1.2 NA water-immersion objective (Nikon, Tokyo, Japan) on an upright microscope (E600FN, Nikon). Images were acquired using an FITC filter cube (Chroma Technology, Bellows Falls, VT), EMCCD camera (DS-Qi1, Photometrics, Tucson, AZ), and Nikon Elements software.

QDs were selected for tracking analysis if they were localized to terminals of isolated bipolar cells or within the IPL of the retinal slices, exhibited a small size (≤4 pixels), and showed intermittent blinking; these criteria are consistent with labeling by a single QD [24]. The location of a QD can be determined with precision exceeding the diffraction limit by fitting the fluorescence profile with a Gaussian point spread function [24,25]. The standard deviation (SD) of displacements exhibited by immobilized QDs suggests pointing accuracies (full-width half-maximum=2.35×SD) of 35 nm for dissociated cells and 55 nm for the retinal slice preparation.

Before tracking, images were smoothed by convolving with a 5 × 5 pixel Gaussian and adjusted to optimize QD contrast. QD position was tracked using NIS-Elements software (Nikon). From the x and y coordinates, we calculated the mean squared displacement (MSD, in μm^2^/s) [26] using the following equation:

$$MSD=\frac{1}{N-n}\overset{N-n}{\underset{i=1}{\sum }}\left({\left[{\text{X}}_{\text{i}+\text{n}}-{\text{X}}_{\text{i}}\right]}^{2}+\;{\left[{\text{Y}}_{\text{i}+\text{n}}-{\text{Y}}_{\text{i}}\right]}^{2}\right)$$

The diffusion coefficient (D) was calculated from the initial slope of the MSD versus time (t) from the origin through the first two data points using data acquired at a rate of 16 ms/frame in dissociated bipolar cells. The value for D was determined with the following equation:

MSD=4DΔt

We calculated the confinement area that a given Ca^2+^ channel traverses within the presynaptic plasma membrane (L^2^) by fitting the data with Equation 3:

$$MSD=\frac{L^{2}}{3}(1-\exp\left[\frac{-12Dt}{L^{2}}\right])$$

For a particle moving freely in solution, all three planes of movement are independent, but movements in a lipid bilayer are constrained to the plane of the membrane. Because z-axis movements could not be tracked, calculated confinement areas and diffusion coefficients represent lower-bound estimates of true values [27]. All data were plotted and analyzed in Prism 4 (GraphPad, La Jolla, CA) and are presented as the mean±SEM.

For the illustrations in the paper, post-image processing was performed in Adobe Photoshop (Adobe Systems Inc., San Jose, CA) to add color and optimize the brightness and contrast.

## Results

**Figure 1:**
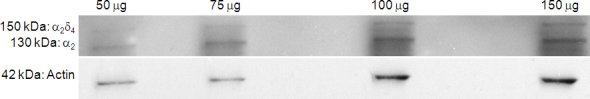
Western blot analysis of the anti-α_2_δ_4_ antibody. Protein lysates from *Ambystoma tigrinum* retinas were resolved with western blot analysis. In this blot, we ran increasing quantities of protein lysate in each lane to optimize the visualization of the presumptive protein bands. Blots were first probed with the anti-α_2_δ_4_ antibody, which revealed two distinct bands at 150 kDa and 130 kDa, indicating the presence of the α_2_δ_4_ and α_2_ proteins, respectively. We then stripped the blot and probed it with an anti-β-actin antibody to assess the successive increase in total protein loaded into each lane.

We tracked the movements of individual L-type Ca^2+^ channels in bipolar cell terminals using QD-based single particle tracking techniques. As described in Methods, streptavidin-coated QDs were attached to individual L-type Ca^2+^ channels by binding a biotinylated secondary antibody to the F_c_ region of a primary antibody against the accessory α_2_δ_4_ Ca^2+^ channel subunit. The antibody was originally developed and shown to be specific for α_2_δ_4_ protein in humans [23]. However, the α_2_δ_4_ amino acid sequence (Ac-KVSDRKFLTPEDEASVC-amide) to which the antibody was raised exhibits excellent homology across vertebrate species, and thus, we predicted that the rabbit anti-α_2_δ_4_ antibody would also bind selectively to α_2_δ_4_ subunits in the tiger salamander retina. Consistent with this, the α_2_δ_4_ antibody labeled photoreceptor terminals in the outer plexiform layer (OPL) and bipolar cell terminals in the IPL [19]. This labeling colocalized with staining for the synaptic vesicle protein SV2 and the α_1_ subunit of Ca_V_1.4 [19]. Labeling was abolished by omitting the primary or secondary antibodies or by including a peptide against which the antibody was raised [19]. We also characterized the antibody with western blot analysis (Figure 1). Western blots using whole salamander retinal lysate and the anti-α_2_δ_4_ antibody produced a band at 150 kDa, as expected for the α_2_δ_4_ subunit. The reducing conditions of the western blot buffer should break the disulfide bond linking the α_2_ and δ_4_ components of the α_2_δ_4_ subunit, and we observed a second band at about 130 kDa, consistent with dissociated α_2_ subunits [23,28]. These data suggest that, like human tissue [23], the antibody exhibits specificity for α_2_δ_4_ in salamander retina.

**Figure 2:**
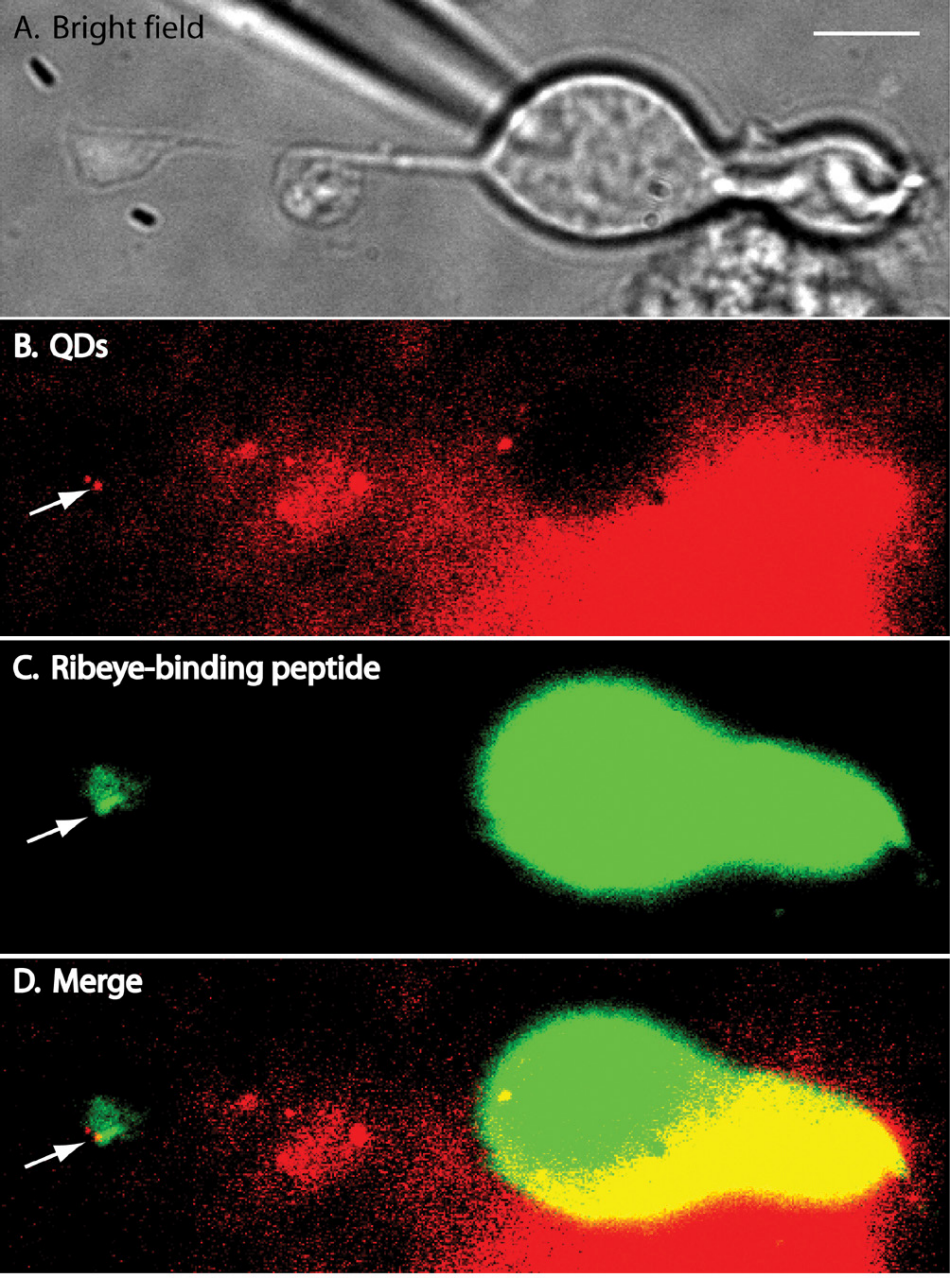
Isolated bipolar cell with Ca^2+^ channels in the synaptic terminal labeled with quantum dots (QDs). **A**: A bright-field image of an isolated bipolar cell. **B**: A fluorescence image of QDs attached to the synaptic terminal of an isolated bipolar cell. The QD adjacent to the arrow was located in the focal plane; the dimmer QD above that one was located in a different focal plane. For this experiment, we used QDs whose emission peaked at 655 nm. The image was averaged from 100 frames acquired at 30 ms/frame. **C**: To label ribbons, we introduced a fluorescent peptide into the bipolar cell through a whole cell patch pipette. The HiLyte 488-conjugated peptide binds selectively to the CtBP domain of the ribbon protein, RIBEYE. **D**: Merged image showing that the bright QD overlapped with a region of bright HiLyte 488 fluorescence in the bipolar cell terminal (arrow).

Figure 2 shows two QDs attached to the synaptic terminal of an isolated retinal bipolar cell. A bright-field image of a bipolar cell is shown in panel A. Figure 2B shows a fluorescence image of 655 nm QDs excited by 561 nm laser light. The QD next to the arrow was located in the focal plane; the second QD was not as bright because its center was located in a slightly different focal plane. In this experiment, we also introduced a fluorescent peptide into the bipolar cell through a whole cell patch pipette to label synaptic ribbons (Figure 2C). We used a HiLyte 488-conjugated peptide that contains a “PXDLS” sequence that binds selectively to the CtBP domain of RIBEYE, the principal ribbon protein [29]. Fluorescence of the brighter QD in Figure 2 overlapped with a region of bright HiLyte 488 fluorescence in the bipolar cell terminal (Figure 2D, arrow). We observed colocalization of QDs with regions of bright HiLyte 488 fluorescence in the terminals of the four other isolated bipolar cells that we tested in this way.

**Figure 3:**
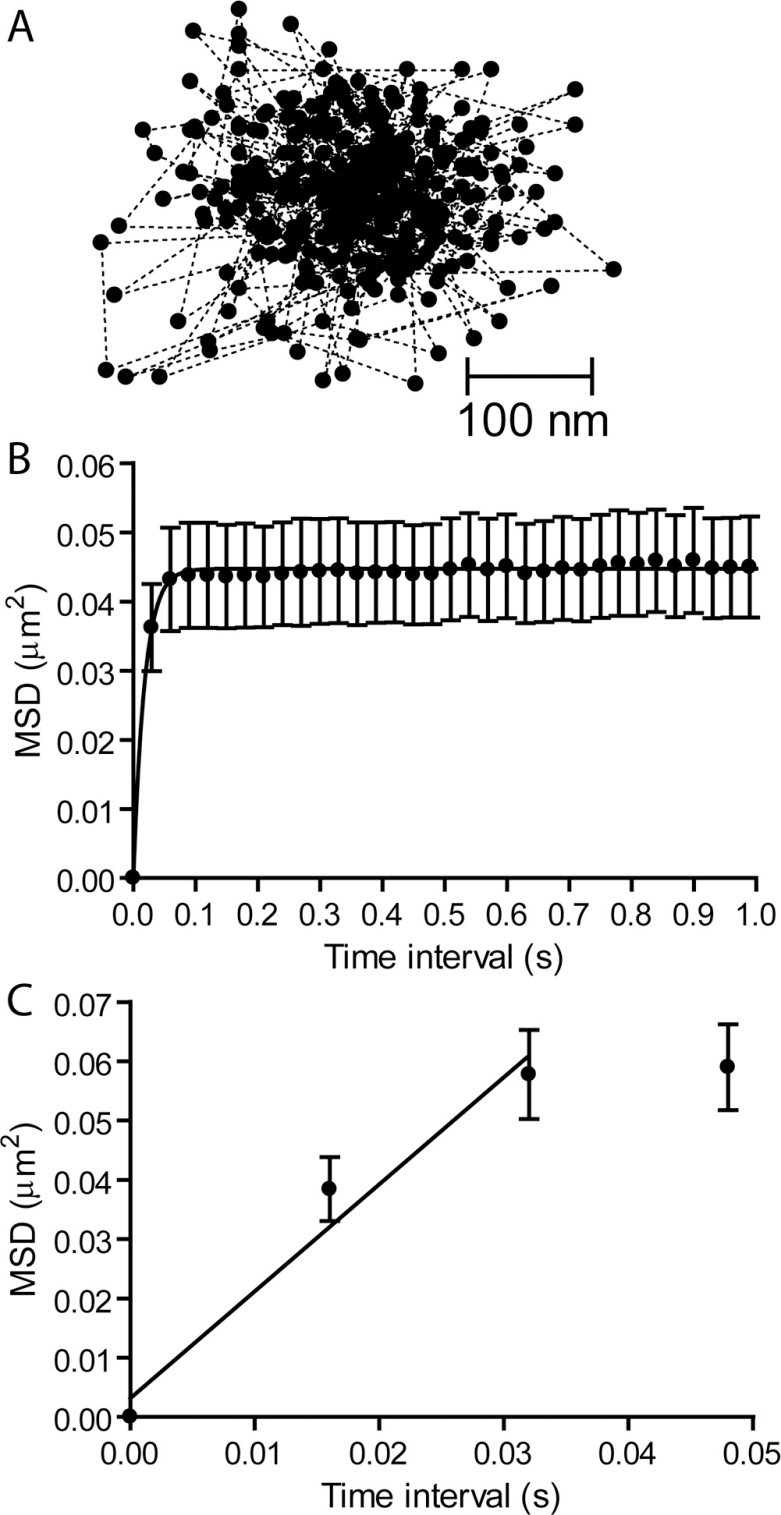
Ca^2+^ channels move within confined domains on the terminals of isolated retinal bipolar cells. **A**: A plot of the trajectory of a single QD showing its position at 400 time points measured every 30 ms. **B**: A plot of mean squared displacement (MSD) versus time interval for individual Ca^2+^ channels in bipolar cell terminals (n=18). Confinement areas were calculated by fitting the data with Equation 3 (L=0.409±0.00479 μm). **C**: A diffusion coefficient of D=0.45±0.24 μm^2^/s (n=9) was determined from data acquired at 16 ms intervals using Equation 2. The straight line shows the fit to the MSD versus time interval relationship from the origin through the first two time points.

In Figure 3A, a trajectory map of the individual positions occupied by a single Ca^2+^ channel was measured at 30 ms intervals for 400 frames. Figure 3B shows the average MSD of the channel positions as a function of the measurement time interval. When visualized on the inverted microscope with a 1.45 NA, 60× objective, the immobilized QDs did not exhibit any measurable MSD. The presence of a plateau in the MSD versus time interval plot indicates that Ca^2+^ channels diffuse within a confined domain [26]. The height of the plateau asymptotically approached L^2^/3 with L=0.366±0.00483 μm (n=24), suggesting a confinement area (L^2^) of 0.134 μm^2^. This is smaller than the confinement areas for Ca^2+^ channels at the rod and cone synapses [19], consistent with the smaller size of bipolar cell synaptic ribbons.

We measured the D by analyzing movements over short time intervals before the channel was confined (Figure 3C). The MSD versus time plot reached a plateau quickly, so we used data acquired at a rate of 16 ms/frame and calculated the D from the initial slope of the MSD versus time from the origin through the first two data points. This yielded a value of D=0.45±0.24 μm^2^/s. Omitting the origin from the linear fit, as was done in an earlier study on photoreceptor Ca^2+^ channels [19], reduced this to 0.3 μm^2^/s.

**Figure 4:**
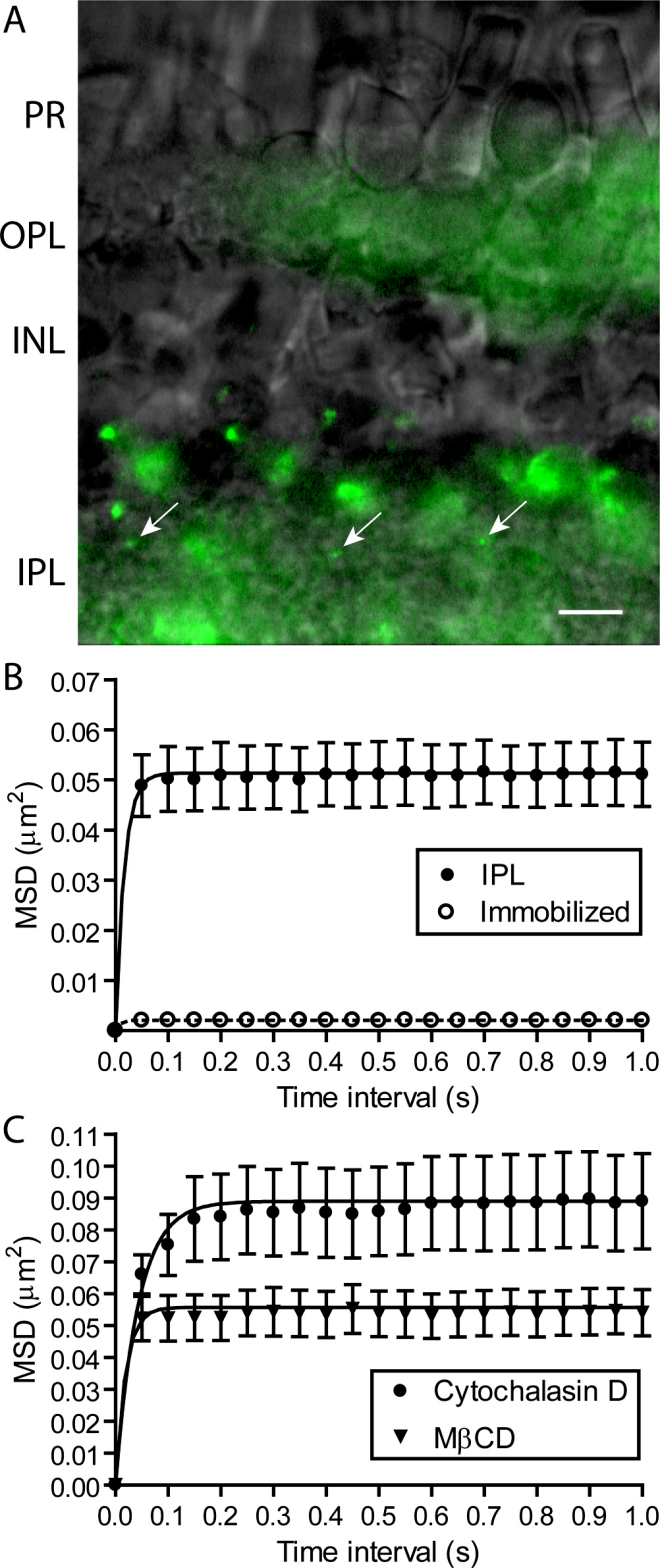
The confinement domains for Ca^2+^ channel movements on bipolar cell terminals in the inner plexiform layer (IPL) were expanded by disrupting actin but not by depleting membrane cholesterol. **A**: Three quantum dots (QDs) labeling individual Ca^2+^ channels in the IPL (arrows). Diffuse autofluorescence is also visible in the inner and outer plexiform layers. Scale bar=10 μm. **B**: MSD versus time interval for movements of individual Ca^2+^ channels in the IPL (n=34; filled circles). Confinement areas were calculated by fitting the data with Equation 3 (L=0.393±0.00237 μm). QDs immobilized in vacuum grease showed a small amount of jitter (L=0.0164±0.00447 μm, n=9; open circles). **C**: Actin disruption with cytochalasin D (20 μM) significantly expanded Ca^2+^ channel confinement domains relative to control (L=0.517±0.00680 μm, n=35, p<0.05; filled circles) but cholesterol depletion with MβCD (10 mM) did not (L=0.409±0.00252 μm, n=16; triangles).

In addition to isolated cells, we also examined the mobility of Ca^2+^ channels ON bipolar cell terminals embedded in the IPL. Figure 2A shows three QDs attached to α_2_δ_4_ subunits in the IPL (arrows). The MSD versus time interval plot yielded a confinement area similar to that found in isolated cells with an average value of L=0.393±0.00237 μm (n=34; Figure 4B). Subtracting the MSD measured for the QDs immobilized in vacuum grease (open circles) reduced this to 0.385±0.00242 μm (L^2^=0.148 μm^2^). Neither the corrected nor the uncorrected values differed significantly from the confinement length L found for isolated bipolar cells (p>0.05).

Cholesterol-rich microdomains and the actin cytoskeleton provide important structural elements for cell membrane compartmentalization [30]. Disrupting actin or removing membrane cholesterol expanded the confinement domains of the Ca^2+^ channels at the photoreceptor ribbon synapses [19]. In bipolar cells, treating retinas for 1 h with cytochalasin D (20 μM) significantly increased the size of the confinement area for the Ca^2+^ channels in the IPL (Figure 4C, circles). Unlike photoreceptors, depleting cholesterol by treating retinas with MβCD (10 mM) did not expand the Ca^2+^ channel confinement areas in the bipolar cell terminals in the IPL (Figure 4C, triangles).

## Discussion

To visualize the movements of bipolar cell Ca^2+^ channels, we attached QDs to individual channels using an antibody-based labeling method. Two concerns about antibody-based QD tracking techniques are often raised: the potential for solvent drag from the fluorophore complex and targeting of a single Ca^2+^ channel with one QD. Regarding the first issue, the viscosity of QDs in aqueous solution is far less than the membrane viscosity, and therefore, QD attachment has only minimal effects on channel mobility [31]. Regarding the second issue, we minimized the possibility that individual QDs might bind multiple Ca^2+^ channels by determining the minimum possible dilutions of primary antibodies, secondary antibodies, and QDs needed to ensure that we labeled only a small number of Ca^2+^ channels [32]. In addition, we used criteria described in the Methods to select Ca^2+^ channels bearing single QDs for analysis.

To attach QDs to Ca^2+^ channels in living, unfixed tissue, we needed a large extracellular domain that can be selectively recognized by the primary antibody. The pore-forming α_1_ subunit of L-type Ca^2+^ channels has only limited extracellular domains [33], and thus, we chose an antibody that targeted the large extracellular domain of the ancillary α_2_δ_4_ subunit. The anti-α_2_δ_4_ antibody [23] used in this study was originally targeted against a human α_2_δ_4_ peptide sequence, but also recognizes the same conserved α_2_ region of *Ambystoma tigrinum* α_2_δ_4_ subunits (Figure 1) and specifically targets the synaptic OPL and IPL in the retina [19]. Although α_1_ and α_2_δ subunits are derived from different chromosomes, α_2_δ subunits are required for the Ca^2+^ channel complex to traffic to the plasma membrane and are thought to remain indefinitely associated with the pore-forming subunit [34]. We found that the radial distributions of Ca^2+^ channel positions were similar among different channels (data not shown) and never saw QDs depart from localized confinement zones, consistent with the idea that α_2_δ_4_ subunits remain associated with other subunits of synaptic L-type Ca^2+^ channels.

The diffusion coefficients for the Ca^2+^ channels in the bipolar cell terminals were 0.3–0.45 μm^2^/s, similar to the diffusion coefficients for the Ca^2+^ channels at the photoreceptor ribbon synapses (0.1–0.2 μm^2^/s) [19]. These values are also within the range of values for the movements of glycine receptors, γ-aminobutyric acid-a (GABA_a_), 2-amino-3-(3-hydroxy-5-methyl-isoxazol-4-yl)propanoic acid (AMPA) and ether-à-go-go (EAG) channels in the extrasynaptic membrane [35-40].

Ca^2+^ channels at bipolar cell terminals were confined within an area of 0.13–0.15 μm^2^. Assuming an active zone width of 200–300 nm [41], this suggests a long axis for Ca^2+^ channel confinement of about 400–750 nm, similar to ultrastructural estimates of the length of the bipolar cell ribbon base (about 400 nm) [21]. Movements of the overlying ribbon could contribute to channel movements and thus the size of the confinement domain, but ribbons in goldfish bipolar cells showed only small movements of about 1 nm/s [6]. Consistent with the larger size of ribbons in photoreceptors, the Ca^2+^ channel confinement domains measured in rods (0.29–0.36 μm^2^) and cones (0.18–0.22 μm^2^) were slightly larger than those of bipolar cells [19,22]. Similar to bipolar cells, Ca^2+^ channel confinement domains in photoreceptors were similar to ultrastructural estimates of the size of the active zone beneath the ribbons [41-44]. At hair cell ribbon synapses, stimulated emission depletion (STED) microscopy of Ca_V_1.3 channel immunostaining revealed an oblong rectangular pattern beneath ribbon-style active zones [12] indicating that Ca^2+^ channels are also organized to mirror dimensions along the base of the synaptic ribbon at a non-retinal synapse.

Disrupting actin expanded the size of the Ca^2+^ channel confinement domain suggesting that protein networks are stabilized at the synapse by interactions with the cytoskeleton [18,45-47]. However, there is no evidence that Ca^2+^ channels bind directly to actin at bipolar cell synapses, and consistent with this, the size of the channel confinement domain and diffusion coefficients were much larger than values obtained for other ion channels that interact directly with actin [48,49]. Unlike photoreceptor synapses [19], removing membrane cholesterol did not alter the Ca^2+^ channel confinement domains, suggesting that cholesterol-rich lipid rafts are not required to confine Ca^2+^ channels near ribbon synapses.

The agreement between measurements of the confinement area made by tracking QD-labeled Ca^2+^ channels and the size of the ribbon determined from ultrastructure in rods, cones, and bipolar cells suggests that single particle tracking techniques may provide a means of measuring the size of the active zone at ribbon synapses in living cells. In cones, fusion of nearby synaptic vesicles increased Ca^2+^ channel displacements and moved channels further away from the center of the confinement area consistent with a brief expansion of the active zone [19]. Expanding the Ca^2+^ channel confinement zone by depleting membrane cholesterol reduced peak release efficiency (i.e., the number of vesicles released per Ca^2+^ channel opening) in cones from three Ca^2+^ channel openings per vesicle fusion event to 4.5 channel openings per fusion event [22]. Similar to cones [16,50], Ca^2+^ channels are close to bipolar cell release sites [5,6]. We therefore expect that changes in the Ca^2+^ channel confinement domain caused by actin disruption or an imbalance between exocytosis and endocytosis might alter release efficiency. Brief treatment with cytochalasin D for 10 min did not significantly alter the total amount of exo- and endocytosis measured with FM1–43 at bipolar cell synapses [47]. Although this would appear to be at odds with the prediction of reduced release efficiency, the total amount of release during a depolarizing test step was also not significantly altered by cholesterol depletion at the cone synapses even though the efficiency of release at the beginning of the step was reduced [22]. Changes in Ca^2+^ channel mobility thus appear to have less influence on the total amount of release and more on the speed and precision of the transmission of light signals through the retina.
